# Network mapping of primary CD34+ cells by Ampliseq based whole transcriptome targeted resequencing identifies unexplored differentiation regulatory relationships

**DOI:** 10.1371/journal.pone.0246107

**Published:** 2021-02-05

**Authors:** Jessica L. Schwaber, Darren Korbie, Stacey Andersen, Erica Lin, Panagiotis K. Chrysanthopoulos, Matt Trau, Lars K. Nielsen

**Affiliations:** 1 Avectas, Toronto, Ontario, Canada; 2 Australian Institute for Bioengineering and Nanotechnology, The University of Queensland, St Lucia, Queensland, Australia; 3 Australian Institute for Molecular Bioscience, The University of Queensland, St Lucia, Queensland, Australia; 4 BlueRock Therapeutics, Toronto, Ontario, Canada; Wayne State University, UNITED STATES

## Abstract

With the exception of a few master transcription factors, regulators of neutrophil maturation are poorly annotated in the intermediate phenotypes between the granulocyte-macrophage progenitor (GMP) and the mature neutrophil phenotype. Additional challenges in identifying gene expression regulators in differentiation pathways relate to challenges wherein starting cell populations are heterogeneous in lineage potential and development, are spread across various states of quiescence, as well as sample quality and input limitations. These factors contribute to data variability make it difficult to draw simple regulatory inferences. In response we have applied a multi-omics approach using primary blood progenitor cells primed for homogeneous proliferation and granulocyte differentiation states which combines whole transcriptome resequencing (Ampliseq RNA) supported by droplet digital PCR (ddPCR) validation and mass spectrometry-based proteomics in a hypothesis-generation study of neutrophil differentiation pathways. Primary CD34+ cells isolated from human cord blood were first precultured in non-lineage driving medium to achieve an active, proliferating phenotype from which a neutrophil primed progenitor was isolated and cultured in neutrophil lineage supportive medium. Samples were then taken at 24-hour intervals over 9 days and analysed by Ampliseq RNA and mass spectrometry. The Ampliseq dataset depth, breadth and quality allowed for several unexplored transcriptional regulators and ncRNAs to be identified using a combinatorial approach of hierarchical clustering, enriched transcription factor binding motifs, and network mapping. Network mapping in particular increased comprehension of neutrophil differentiation regulatory relationships by implicating ARNT, NHLH1, PLAG1, and 6 non-coding RNAs associated with PU.1 regulation as cell-engineering targets with the potential to increase total neutrophil culture output. Overall, this study develops and demonstrates an effective new hypothesis generation methodology for transcriptome profiling during differentiation, thereby enabling identification of novel gene targets for editing interventions.

## Introduction

Neutrophils are the body’s most abundant white blood cell and the first line of immunological defence against pathogens. When a person has an absolute neutrophil count less than a 500 per μL (a state defined as severe neutropenia) they are highly susceptible to infection with associated morbidity and mortality [1, 2]. Chemotherapy is one cause of neutropenia, as treatment ablates all rapidly dividing cell types including a patient’s own progenitor immune cells. Following chemotherapy, patients are neutropenic throughout the period of time required to replenish the ablated neutrophil progenitor population, with a median neutropenic time of 23 days for a single high dose chemotherapy and increasing to over 39 days for multiple chemotherapy treatments [3–5].

This immune-compromised state accounts for the majority of chemotherapy related deaths [4, 5], and there are different therapeutic options to address it. Treatment with granulocyte colony stimulating factor (GCSF) can shorten the time to recovery, although there is still a neutropenic lag period [6–8]. In a leukemia setting haematopoietic stem cells (HSCs) can be transplanted after high dose chemotherapy for haematopoiesis reconstitution in a process termed haematopoietic stem cell transplantation (HSCT). An alternate method to alleviate neutropenia is through *ex vivo* differentiation of neutrophils into mature phenotypes first, prior to HSC transfusion [9–12] which broadens protective applicability of the method to all chemotherapy patients, as HSCTs are specific to leukemia treatment, but *ex vivo* neutrophil transient protection may be applied across all chemotherapy treatments that incur neutropenia. For example, a robust 15-day *ex vivo* neutrophil production protocol from CD34+ progenitors has been developed, and the mature cell product (Theraphils) has successfully passed standard quality tests for neutrophils [11, 12]. The assay started with a proliferating granulocyte progenitor phenotype of improved homogeneity over the broad CD34+ progenitors from which a causal progression of transcriptional regulators may be linked in a time-informative manner.

However, while success has been seen with both HSCT and *ex vivo* transient treatment, the cost per output remains too high to become broadly implemented in health care systems. For example, given the current expansion potential of umbilical cord blood (UCB), 1 cord blood unit is adequate for either one HSCT or neutrophil transfusion [9, 11], meaning current expansion protocols are not efficient enough to be medically translatable at current expansion numbers and transient protection can only be administered as prophylactic.

One avenue for increasing expansion potential would be to define a succession of regulatory entities to enable a better understanding of the regulatory mechanisms driving differentiation, neutrophil commitment, and lineage cell state transitions. However, although the molecular components of neutrophil maturation that define cell surface marker profiles and granule development are well established, many elements of the pathway in the intermediate phenotypes between the granulocyte-macrophage progenitor (GMP) and the mature neutrophil phenotype are unclear, with the exception of a few master transcription factors (PU.1, CEBPA, CEBPE, CEBPD, and GFI). Consequently, the causative relationships driving the transitions from the stem cell to mature phenotype remain undefined.

As culturing approaches have been explored extensively, future methods towards improving output would benefit from comprehension of the regulatory elements governing the underlying processes of neutrophil differentiation and commitment. Given this, we hypothesized that a better understanding of the regulatory mechanisms driving differentiation—and specifically neutrophil commitment and lineage cell state transitions—would provide the opportunity to improve Theraphils using cell engineering, and thereby achieve increased functional cellular output. Within this scope, we undertook a hypothesis-generating study to delineate potential transcription factor activation networks during lineage progression of neutrophil differentiation using an improved primary cell culturing and sampling technique, combined with measurement of global gene expression during neutrophil differentiation. To assess this question the Ampliseq RNA method was utilized, which represents a novel targeted-resequencing whole transcriptome interrogation method that employs a over 20,000 quantitative reverse-transcriptase PCR assays multiplexed together to determine the expression profile of distinct human RNA targets. In particular, we sought to assess this assay for its sensitivity in detecting expressed targets, particularly when working with low input and low quality sample template, and its utility in identifying network relationships across a sample time-series.

To assess Ampliseq’s capability for both detecting known relationships and for discovery-based analyses, we performed an analysis of the capability of whole-transcriptome Ampliseq panel. Specifically, time series analysis was conducted during the neutrophil differentiation of a granulocyte progenitor (GMP) cell population. Results obtained with Ampliseq were then validated against expectations based on the literature, digital PCR based targeted transcriptomics and mass spectrometry proteomics analysis, with the goal of both validating Ampliseq as an appropriate tool for the sensitive and specific detection of regulators and their networks.

## Materials and methods

### Culture preparations

#### Cord blood isolation

Fresh human umbilical cord blood (UCB) was obtained with informed consent following institutional ethics review and approval. This project was approved by the Royal Brisbane and Women’s Hospital Human Research Ethics Committee (HREC/14/QRBW/426). Cord blood was collected with written consent from mothers of newborn babies. CD34+ cells were isolated from UCB within 24h of collection from full term pregnancies at the Royal Brisbane and Women’s Hospital. Briefly, whole UCB was separated by density gradient centrifugation on Ficoll-Paque Plus (GE Healthcare Life Sciences), and the mononuclear cell fraction collected for magnetic isolation of CD34+ cells by MACS (Miltenyi Biotech) according to the manufacturer’s instructions. Cells were cryopreserved in 1mL volumes of FBS and 10% DMSO and frozen slowly at -1°C/min using a polycarbonate container with isopropyl alcohol at -80C.

#### Preculture

Cryovials were thawed quickly in a 37°C water bath and diluted in 10mL StemLine^®^II medium (Sigma-Aldrich) before centrifugation at 200xg. Thawed cells were cultured in StemLine^®^II medium (Sigma-Aldrich) supplemented with 100ng/mL of SCF (Amgen), 2mM GlutaMAX^™^ (Invitrogen), and antibiotic-antimycotic solution (penicillin/streptomycin/fungizone; Invitrogen). Cultures were incubated at 37°C with 5% CO_2_. Precultures were seeded at variable densities and cultured for 2 days before proceeding with analysis.

#### Cell preparation and Fluorescence Activated Cell Sorting (FACS)

Pre-cultured cells were incubated with FcR Blocking reagent (Miltenyi Biotech) for 10 minutes at 4°C. Thereafter, cells were labelled with fluorochrome-conjugated monocolonal antibodies (BD Biosciences unless mentioned otherwise) PE-conjugated anti-IL-3Rα; PerCp-Cy5.5-conjugated anti-CD34+; FITC-conjugated anti-CD45RA; A488-conjugated anti-CD38 (BioLegend); PacificBlue-conjugated anti-Lineage (CD3/14/16/19/20/56) and anti-CD11b (BioLegend); BV421-conjugated anti-CD10 and anti-CD7. Dead cells were excluded by Draq7 (Cell Signaling Technology).

All experiments were performed using a BD Influx^™^ fluorescence activated cell sorter using Sortware 1.0.1.6 software. Instrument settings were: sheath pressure of 12 PSI, 100um nozzle, 28 KHz drop drive frequency, sorting with a "1.0 single cell" sort mode resulting in a sort efficiency of ≥85%. Isotype controls were not used in assessing progenitor cell isolation [13, 14]. Background staining and fluorescence compensation was performed using a control sample stained with a single monoclonal antibody.

Gated IL-3Rα^lo^CD45RA+ GMP populations were isolated with 1.2x10^5^ cells sorted for RNA extraction (Day 0) and the remaining GMPs were used for initiating granulocyte cultures.

#### In vitro granulocyte differentiation culturing assays

Cultures were initiated at a density of 1 x 10^4^ cells/mL in a volume containing StemLine^®^II medium (Sigma-Aldrich) supplemented with 100ng/mL each of Stem Cell Factor (SCF) (Amgen), Granulocyte Colony Stimulating Factor (G-CSF) (Amgen) and a Thrombopoietin (TPO) peptide mimetic (AusPep), and 2 mM GlutaMAX^™^ (Invitrogen) and antibiotic-antimycotic solution (penicillin/streptomycin/fungizone, Invitrogen). Cultures were incubated at 37°C with 5% CO_2_ in 90% humidity.

#### Sampling

Samples were collected at 24 hour time points. 1.2x10^5^ cells were collected in triplicate for each population-based Ampliseq transcriptome analyses and varying amounts of cells were harvested depending on culture expansion for proteomic analyses (1x10^6^ for analysed samples) (Table 1). Cells for proteomic analysis were washed, pelleted and stored at -80°C until sample preparation. RNA extraction for Ampliseq analysis is described in the next section.

**Table 1:** Cell number contained in samples across time for both Ampliseq transcriptome analyses and proteome analyses.

Sample Days	Transcriptome Cell #	Proteome Cell #
1–2	120,000	100,000
3–5	120,000	500,000
6–9	120,000	1,000,000

Sample sizes increased for proteome analyses as cultures expanded in later sampling time points.

Based on the high variability and/or absence of the housekeeping/control proteins, data of sampling days 1–5 were removed from further proteomics analyses as unreliable (S7 Fig). We hypothesise that the protein extraction protocol followed here is not optimal for so few cells. As the transcriptome samples were sound for all samples, we proceed with days 0–9 for transcriptome and days 6–9 for proteome analyses.

### Sample preparation

#### RNA isolation and DNase cleanup

At 24 hour time points, 1.2x10^5^ cells were harvested for total RNA purification using the Qiagen Rneasy Plus Micro Kit. An off column 1U TURBO^™^ Dnase digestion (Ambion) was followed by a Zymo-5 column clean up kit (Zymo Research). Sample quantity and quality were assessed using the Qubit (ThermoFisher Scientific) and a Bioanalyzer (Agilent) run respectively. All sample RNA Integrity Numbers (RINs) were 9 or above.

#### Reverse transcription and library prep

Reverse transcription was performed with 30ng of RNA using SuperScript^®^ Vilo^™^ (Invitrogen) in 10uL reaction volumes. The initial 25°C 10 minute incubation was followed by an extended 2 hour incubation at 42°C. The reactions was terminated with a 5 minute incubation at 85°C.

Libraries were prepared using Ion Ampliseq Transcriptome Human Gene Expression Kit. Samples were prepared in triplicate as technical replicates and spread over 3 sequencing runs on the Life Technologies Proton sequencer using the Ion PI Templating and Sequencing V3 200 kits, approximating 80 million reads/run with the number of reads split roughly equally across the samples. Probe mapping was performed using Torrent Suite Software version 4.4.3 against the vendor reference sequence for the Ampliseq Transcriptome (hg19_ampliseq_transcriptome_ercc_v1.fa).

#### Proteomics: Swath sample preparation

Cell pellets were thawed and resuspended in 150uL ice cold 8M urea and 50mM ammonium bicarbonate buffer containing protease inhibitors (2 mM sodium orthovanadate, 5mM sodium fluoride, 5mM sodium pyrophosphate, 5mM b-glycerophosphate, 2 mg/mL Aprotinin and 1 mg/mL Leupeptin). Cells were further disrupted by passaging through a 22G needle 5 times. Complete cell lysis was achieved by 3 x 1 min sonication sessions with 1 min break intervals on ice. DNA and RNA were digested by 5μL of RnaseA and DnaseI (Thermo Fisher Scientific) for 15 min at room temperature. After centrifugation at 16,000 g for 10 min at 4°C the supernatant was transferred to new Eppendorf tubes and the final protein concentration was determined using a BCA Protein Assay Kit (Thermo Fisher Scientific). Proteins were then reduced by 5mM dithiothreitol (DTT) for 45 min at room temperature and alkylated by 25mM iodoacetamide (IAA) at room temperature for 30 min. Total protein was retrieved upon precipitation by the addition of 1 ml of ice cold acetone and incubation for 2 hours at -20°C. The samples were centrifuged at 1000g for 10 min and the supernatant was discarded. Pellets were then resuspended in 150 μL ammonium bicarbonate (AMBIC) and digested with trypsin (Trypsin Gold, Promega) at 37°C for 16 hours according to the manufacturer’s protocol. Trypsin digests were centrifuged at 16,000 g at 4°C for 10 min. The pH was adjusted to 3 by the addition of 5% formic acid (FA) in 5 μL steps. 150 μL of 0.1% FA was added to the peptide digests before they were desalted with C18 ZipTips (Millipore). In detail, the C18 ZipTips were equilibrated with 100% acetonitrile (CAN) and 0.1% FA sequentially. The samples were then loaded, washed with 0.1% FA and eluted with 50% ACN. Prior to MS analysis, the eluted peptides were dried in a vacuum concentrator and resuspended in 100 μL 0.1% FA resulting in a 1μg/μL final peptide concentration. The total amount of 4 and 0.5μg of protein were used for the IDA and SWATH analysis respectively.

A sequential window acquisition of all theoretical fragment ion spectra (SWATH) peptide reference library was generated based on peptides from all biological samples that were combined in a pooled sample and were chromatographically fractionated. In more detail, peptides were separated using an Agilent Macroporous Reversed-Phase C18 (mRP-C18) High-Recovery Protein LC Column at 0.75 ml/min on an Agilent 1200 chromatographer according to the manufacturer’s protocol. 54 fractions were collected and were pooled to give 14 final fractions based on the UV chromatogram. They were then dried in a vacuum centrifuge, resuspended in 100 μL of 0.1% FA and were desalted as described above. Information-dependent acquisition (IDA) of the fractions was used for the construction of the SWATH reference library.

#### Proteomics: LC-MS/MS analysis

For the proteomic discovery and sequential window acquisition of all theoretical fragment ion spectra mass spectrometry (SWATH MS) analyses, samples were separated on a Shimadzu Prominence nano U-HPLC system as reported by Kappler *et al*. [15], with some modifications. A desalting stage with the Agilent C18 trap (0.3 x 5 mm, 5 μm) at 30μl/min flow rate was run for 3 min. The samples were then loaded, at a 1μl/min flow rate, on a Vydac Everest C18 (300 A, 5 μm, 150 mm x 150 μm) column. The peptides were eluted from the column with a linear gradient of 10–60% solvent B (80% acetonitrile/ 0.1% formic acid v/v) over 75 min. The LC was coupled with a 5600 TripleTOF^™^ mass spectrometer (ABSciex) equipped with a Nanospray III interface. Gas 1 and curtain gas were set at 10 and 30psi respectively while ion spray floating voltage was 2700 V. For the discovery analyses, the mass range was set across m/z 350–1800 for 0.5 sec followed by IDA on high sensitivity mode of the top 20 precursors with intensity greater than 100 counts across m/z 40–1800 for 0.05 sec. The collision energy was set at 40 +/- 15 V. For the SWATH analyses, a set of 32 overlapping, variable Q1 isolation windows (1 Da overlap) were used to scan across the mass range 400–1200 Da [16]. Each window was scanned for 0.1 sec and the collision energy was automatically assigned by Analyst software.

#### Digital PCR

High-throughput quantitative Evagreen ddPCR technical triplicate samples for ACNT4, POL2, PU.1, and MEIS1 were prepared for each time point. 20 ng RNA from each time point used for reverse transcription using SuperScript^®^ Vilo^™^ (Invitrogen) in 10uL reaction volumes. The initial 25°C 10 minute incubation was followed by an extended 2 hour incubation at 42°C. The reactions were terminated with a 5 minute incubation at 85°C. cDNA samples were diluted 1 in 40 with UltraPure water (Invitrogen) and 1 uL was added to each QX200 ddPCR EvaGreen supermix with corresponding human forward and reverse primers at 200nM (POL2: forward 5’- ATG GTT CTA CAA ACA GCC AGT ACC CAG/reverse 5’- AGA CTT GGT CTG GTT GAA GAT AAC AAT GTC, PU.1: forward 5’-GAG GTG TCT GAC GGC GAG GC/ reverse 5’-TGC GGA GCA GGT CCA ACA GG, and MEIS1: forward 5’-CGT AAT GGA CGG TCA GCA ACA/ reverse 5’-CCC CTC CAT GCC CAT ATT CA). PrimePCR^™^ SYBR^®^ Green Assay validated human forward and reverse primer, ACTN4 was added at 250nM (Bio-Rad). Final reaction volumes were 22uL. Controls of no template and no template or primers were included in triplicate.

Droplets were created using the Automated Droplet Generator and PCR run using a C1000 Thermal Cycler (Bio-Rad) with the following thermal cycling protocol: enzyme activation at 95°Cfor 10 minutes, followed by 40 cycles of denaturation at 95°C for 30 seconds and annealing/extension at 50°C for 60 seconds, and a final signal stabilization cycle of 4°C for 5 minutes and 90°C for 5 minutes. Thereafter, a QX Bio-Rad Droplet Reader was used for signal detection and quantification.

### Data analysis

#### Ampliseq

Low count genes were removed. Of the 20802 Ampliseq targeted genes, 12,963 passed the threshold of having at least 2 samples expressing more than 1 read. The sample variance was stabilised using an rlog transformation as a global-sample normalization [17]. Variables were then transformed to a scale between 0 and 1, retaining rank order and the relative size of separation between values. The raw counts can be found in S1 File. The data was normalized using DEseq2 and then rlog transformed as described below. For.bam files, please contact the author of correspondence.

#### Clustering (cluster 3), visualization of clustering (TreeView) and GO-analyses

DESeq2 rlog transformed data was Euclidean distance average linkage clustered using Cluster 3.0 software [18]. Prior to clustering, both genes and arrays were median centered and then normalized such that the sum of the squares of the row and column values were approximately 1.

The result of the hierarchical Euclidean distance average linkage clustering calculation was displayed in a heatmap as a gene and sample dendrogram. The dendrogram was colored to represent a > 0.8 correlation threshold [19].

GO Enrichment analyses were run on clusters containing PU.1, CEBPΕ, CEBPD, and GFI1 [20]. Revigo was used to visualize the GO enrichment analysis results with the box size reflecting p-values [21].

Transcription factor binding motif enrichment analysis was performed on each cluster separately through the TFM-Explorer web interface, using weight matrices from JASPAR and TRANSFAC and a range of -10 000 to +500 relative to TSS.

#### Proteomics: Bioinformatics analysis

MS/MS data from IDA, and fractionated IDA were analyzed using the Paragon Algorithm from ProteinPilot v4.5 (ABSciex). The 14 fractions’ IDA were analysed simultaneously using ProteinPilot. Reviewed Protein sequences for Homo sapiens were downloaded from the Universal Protein Resource (uniprot_sprot.fasta.gz file in http://www.uniprot.org/downloads) containing 70236 proteins. Search parameters were set to include trypsin digestion, MMTS as cys-alkylation, FDR analysis, and “thorough” settings. Only proteins identified with at least two peptides with more than 95% confidence score and false discovery rate lower than 1% were included in the statistical analysis (584). MS/MS data from SWATH were analysed with the SWATH processing script within PeakView v.1.2 (ABSciex). The discovery data of all the fractions and the pooled sample were used as the spectra library for protein identification. The sum of the fragment ion peak areas of each peptide were calculated by PeakView v.1.2 (ABSciex) and were used for quantification.

Manual, targeted interrogation of the raw SWATH data was performed using Skyline v2.6 (http://proteome.gs.washington.edu/software/skyline/). A minimum of 10 overlapping y+ and b+ transitions and 2 peptides were used for the identification of each peptide and protein respectively. Peptides were quantified by summing the peak areas of at least 4 transitions. Protein abundance was calculated as the sum of at least 2 peptides. Normalisation for total protein loading variation was performed by dividing each protein’s abundance by the sum of total integrated peak areas per sample (S2 File). For raw data files, please contact the author of correspondence.

#### Digital PCR data analysis

QuantaSoft analysis software (Bio-Rad) was used to determine the total number of droplets read. A threshold for defining positive droplets was determined by manually setting the threshold 1500 above control samples, defining the number of positive droplets/uL, an absolute number of transcripts per microliter. These results were multiplied by the total sample reaction volume of 22uL for a final absolute quantification of a given target.

The normalization factor for each sample was determined using the reference genes by determining the geometric mean from the replicate average of total detected transcripts in the reaction. The normalizing factor was computed for each sample by setting the total variances equal to zero. This normalization factor was applied to the total detected transcripts of each TF across the time series.

## Results

### Granulocyte-macrophage progenitor cell culture

To assess the progression of transcriptional regulation events this study employed an *ex vivo* expansion system which enabled cell population sampling at 24-hour time points over a 9 day period to infer correlated expression patterns. To improve the homogeneity of the cell culture starting material, granulocyte-primed progenitors (granulocyte-macrophage progenitors (GMPs)) were isolated from pre-cultured CD34+ cells; we were therefore able to remove other cell types such as multiple lineage-biased progenitors (megakaryocyte-erythroid progenitor (MEP) and common lymphoid progenitor (CLP)) as well as the more quiescent and unbiased common myeloid progenitor (CMP). However, the GMP phenotype is still heterogeneous as it contains cells in various states of quiescence. To further increase the culture homogeneity, a 2-day pre-culture period was used prior to GMP isolation in order to bring all the cells into a proliferative state. A single umbilical cord biological sample was used for this study, with three technical replicates at each time point.

### Performance validation of Ampliseq data

**Fig 1:**
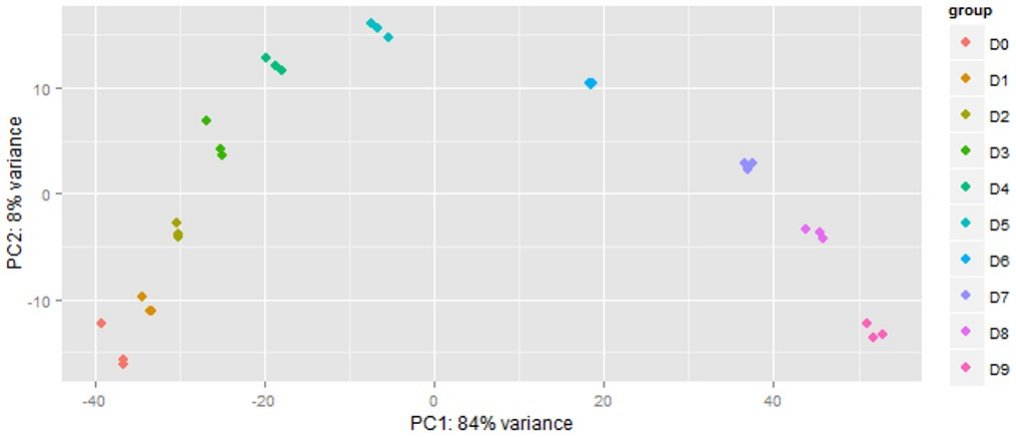
PCA plot of samples shows clustering of three technical replicates per timepoint, together with a trend associated with the sampling day. PC1 at 84% variance is largely comprised of factors driving cell structure, immunological and inflammatory responses. The second principal component rotates around a gene expression profile shift at day 5. Components contributing to the variation captured in PC2 include plasma and cellular membrane, extracellular matrix, and signalling. These two vertices account for over 90% of the sample variance. For and the top 575 loadings of PC1 and PC2, refer to S3 File.

Of the 20802 Ampliseq targeted genes, 12964 passed the threshold of having at least 2 of the three technical replicates for a single time point expressing more than 1 read. The sample variance was stabilised using an rlog transformation in the DESeq2 package [17] as a global-sample. The data transformed data showed good clustering of the experimental replicates using principle component analysis and suggested a high technical replication capacity of the Ampliseq RNA method (Fig 1). Notably, the first principal component contained 84% of the variance and was represented largely by genes related to cell structure and immunological/inflammatory responses. The second PCA component representing 8% of the variance which was attributed to genes related to plasma and cellular membrane, the extracellular matrix, and signaling which are up regulated and then down regulated (or vice versa) around day 5.

To determine whether suitable reference housekeeping genes with the Ampliseq RNA dataset could be identified, we examined the expression patterns of the largest subunit of RNA polymerase 2 (*POL2*) and *ACTN4*. The largest subunit of *POL2* has been identified by a previous study as a stable reference gene and was confirmed to have stable low expression across the Ampliseq time series data [22] (S2 Fig). Similarly, *ACTN4* was previously identified as being a potential reference gene [23], and showed stable expression across the time series transcriptome (S2 Fig) and proteomic analyses herein.

Next, droplet digital PCR (ddPCR) was used as a quantitative gene expression tool to validate gene expression levels as determined by the Ampliseq RNA method. In particular, two well characterized granulocyte-related transcription factors with opposite expression trends were selected for ddPCR validation. The transcription factor MEIS1 is expressed in stem and progenitor cells and is downregulated during granulocyte differentiation [24, 25]; and PU.1 (SPI1) is a known master transcriptional regulator that drives myeloid cell differentiation and mediates induction of myelomonocytic genes [26]. A Pearson correlation was used to compare normalized ddPCR and Ampliseq time series analyses for *MEIS1* and *PU*.*1*, which gave an r^2^-value of 0.985 and 0.918, respectively.

**Fig 2:**
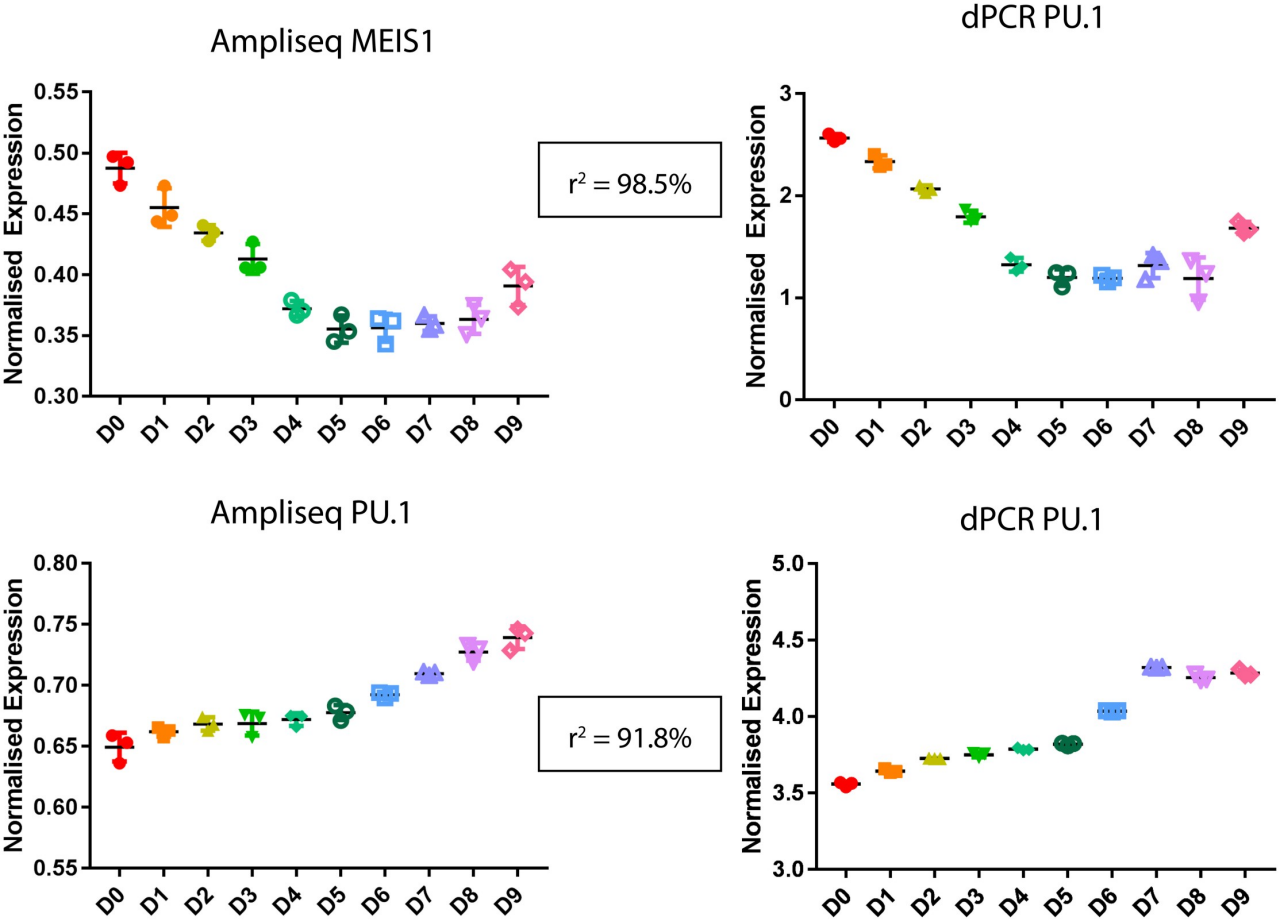
Digital PCR was used as a validation approach for 2 transcription factors (MEIS1 and PU.1). The expression profiles for each target overlap for each method: Ampliseq and ddPCR, supporting Ampliseq as a sound method for transcriptome analyses. *MEIS1* is expressed in stem and progenitor cells and shows decreased expression over the time series and comparatively, *PU*.*1* is increased in expression as it is a known master regulator of the myeloid lineages.

A scatterplot comparison of ddPCR and Ampliseq RNA expression values plotted against each other similarly showed a strong linear correlation between the two methods for the selected genes (S2 Fig). For reference, the Ampliseq sequencing library depth is shown in S2 Fig. From these results, it can be concluded that Ampliseq presents comparable resolution to ddPCR for the expression trajectories of selected transcription factors and reference genes (Fig 2).

Prior to discovery-based analyses, known targets were analyzed in both transcriptome and proteome samples to ensure that the differentiation progressed as expected, as well as to assess the performance of Ampliseq method. Known factors comprising cell surface receptors and granules were used for this analysis based on their defined expression trajectories for the neutrophil differentiation pathway. For example, as granulocyte colony-stimulating factor (GCSF) is used to stimulate CD34+ cells to neutrophils, the *GCSF* receptor (*GCSFR*) is expressed early on and upregulated during the differentiation progression [27]. Similarly, *CD11b* (*ITGAM*) is upregulated later in the myelocyte stage and transcriptionally is upregulated ~ day 5 [28]; *CD16* (*FCRγIII*)expression starts at the metamyelocyte stage and increases in expression until highly present on the neutrophil phenotype [29, 30]; and *CD10* (*MME*) expression only comes up in expression on the final, mature neutrophil [29, 30].

**Fig 3:**
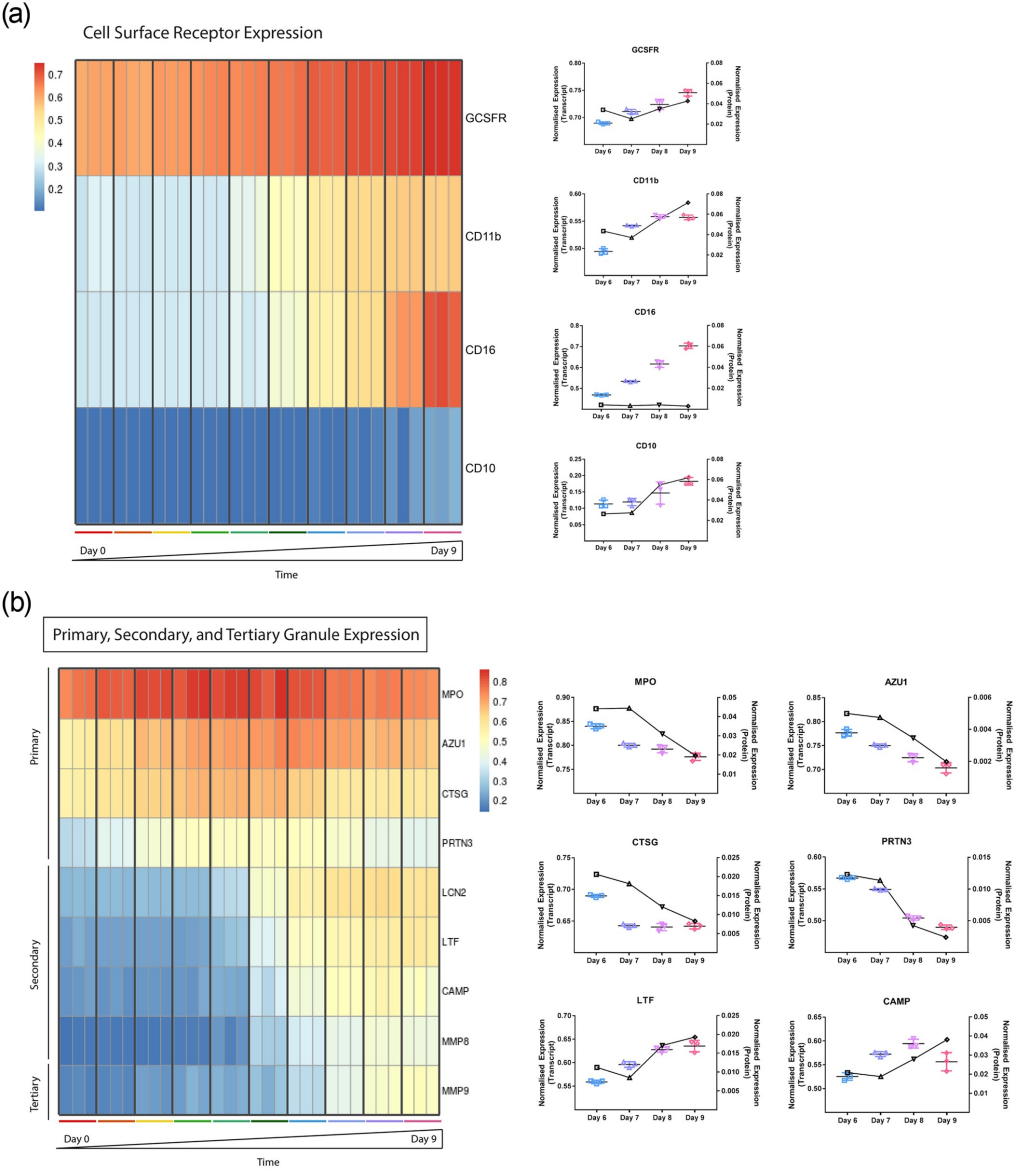
(a) Cell surface receptor transcript and protein expression profiles. The transcript expression of four receptors known for increased expression during neutrophil differentiation is shown as a heatmap on the left from day 0 to 9 and the protein expression profiles from day 6–9 on the right. The receptor CD16 increased progressively from day 6–9 in the transcriptome dataset, however, maintained low, invariable protein expression. This also may be an artifact of cell surface receptors being difficult to isolate. The heatmap values are plotted post-DESeq2 normalization and as the rlog transform to a scale between 0 and 1, retaining rank order and the relative size of separation between values. (b) Cell surface receptors, granules, transcription factors and protein expression profiles. Cell surface receptors, granules, transcription factors and protein expression profiles were used to assess the performance of Ampliseq in capturing defined factors in the granulocyte progression. The granule transcript expression profiles from day 0 to 9 are shown as a heatmap on the left and for days 6–9 granule protein expression profiles on the right. The expected expression timing and progression was captured by Ampliseq and a general trend was maintained between transcript and protein expression. The heatmap values are plotted post-DESeq2 normalization and as the rlog transform to a scale between 0 and 1, retaining rank order and the relative size of separation between values.

While transcriptome analyses were run in triplicate, there were only enough cells remaining for one protein replicate at the high requisite of 1 million cells. Therefore, protein data was collected as a basic check for consistency between transcription and translation and the general trend was verified. For example, comparison of Ampliseq RNA expression levels to protein expression levels for these cell surface receptors observed notable trajectory overlap between receptor transcript and protein expression (Fig 3a). An increase in *CD11b* expression was seen in both transcriptome and proteome profiles, however, the latter lagged by a day, being upregulated on day 8. This delay may be due to time required for protein translation. The exception to this was *CD16* which maintained a low, steady protein expression profile while the transcript expression profile showed a clear increase in expression starting on day 6. This may be an artifact of a delay due to translation, or an example of difficulty in isolating proteins expressed on the cell surface, as cell surface receptors are acknowledged as difficult to isolate for proteomic analyses [31]. Another potential consideration could also be that the Ampliseq RNA method targets a single exon pair (similar to standard qRT-PCR), and the protein level determined by proteomic analyses could represent translation of a specific splice isoform different from that being assayed by the Ampliseq RNA method. To test this possibility that different splice variants of CD16 were represented in AmpliSeq and mass spectrometry analysis, qRT-PCR for additional exon pairs may be performed. Overall the Ampliseq RNA appeared to capture the anticipated progression of cell surface receptor expression effectively.

The presence, timing, and progression of the sequential expression of primary, secondary and tertiary granules were also used to further assess the data. Azurophilic, primary granules may begin to appear in the late myeloblast, but are a characteristic feature of the promyelocyte and contain various antibacterial proteins, proteolytic enzymes and myeloperoxidase [32–34]. In the myelocyte, the highly numerous secondary granules are expressed, containing the adhesive proteins lactoferrin and integrins. And upon the development of the fully functional phagocytic band phenotype, the tertiary (gelatinase) granules are upregulated, with a maintained primary to secondary granule ratio of 1:2 [35]. This expected progression was seen with primary granules starting to be expressed at day 0 and the gelatinase, tertiary granule, MMP9, in the final days of culture (Fig 3b). Additionally, general trends were maintained between gene and protein expression (of the proteins retrieved upon manual interrogation of the SWATH data).

### Proteomic profiles

**Fig 4:**
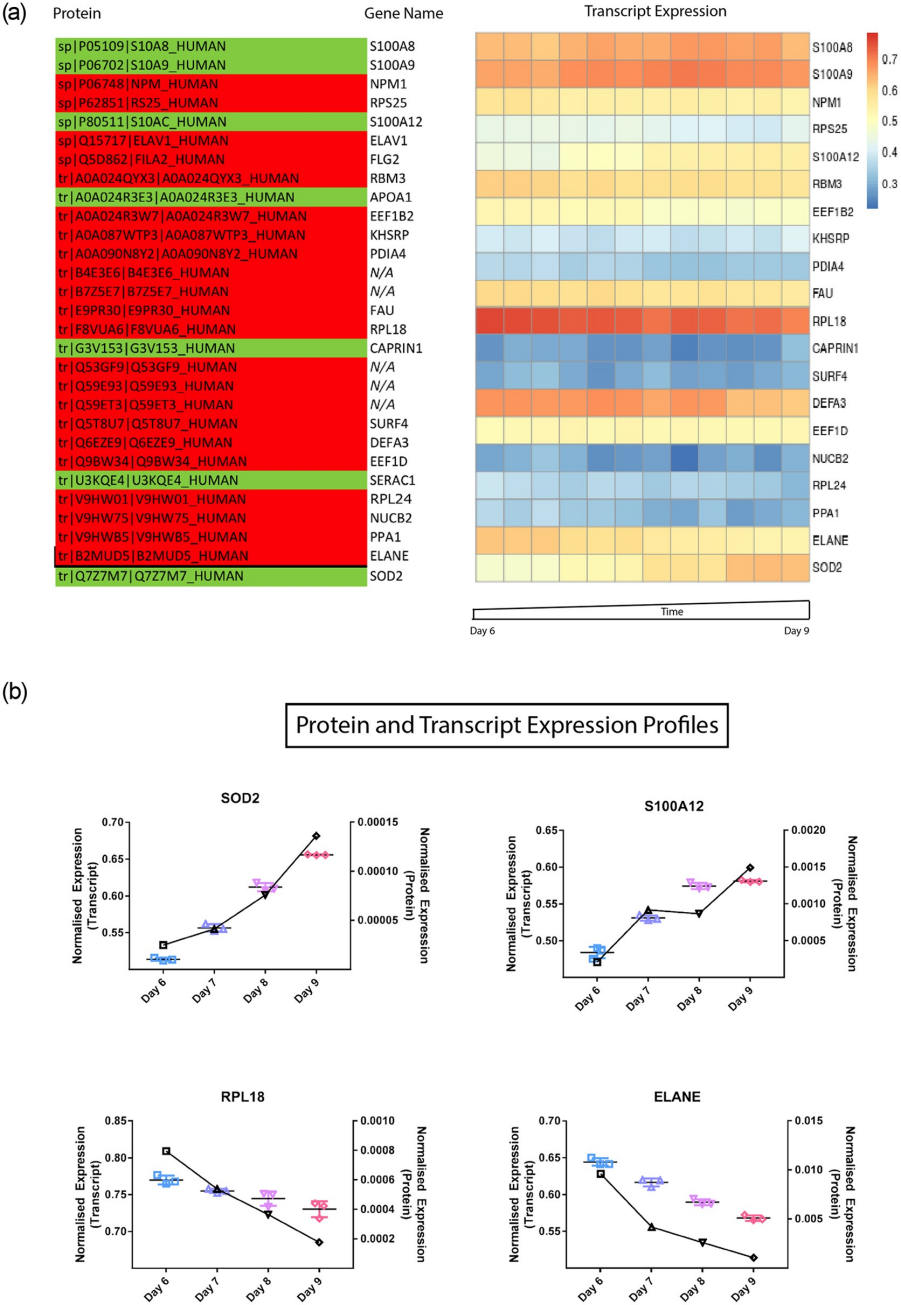
(a) The most variable proteins from the SWATH analysis from days 6–9 of the differentiation time series. An increasing trend is highlighted in green and a negative trend in red. The corresponding gene name is provided next to the protein column and the associated transcript expression was displayed in a heatmap from day 6–9 for comparison. ELAV1(HUR) had no Ampliseq probe to detect it and FLG2, APOA1, and SERAC1 did not pass the initial filtering expression threshold. The heatmap values are plotted post-DESeq2 normalization and as the rlog transform to a scale between 0 and 1, retaining rank order and the relative size of separation between values. (b) Overlaid protein and transcriptome expression profiles selected from the most variable proteins from the SWATH analysis from days 6–9 of the differentiation time series. Transcript expression profiles are shown in colour and in triplicate and proteomic data is overlaid in black, as a line graph. Graphs are labelled with the associated transcript name.

Given that the previous analysis evaluating cell surface receptor, granule, and transcription factor and protein expression profiles showed good concordance, we next undertook a protein discovery analysis. In the top 100 most variable (coefficient of variation >50%) proteins from the SWATH analysis, thirty proteins displayed a prominent upward or downward expression trend. Only 7 of these had an increasing trajectory (green), with the remainder decreasing (red) (Fig 4a). These proteins were annotated for the corresponding gene name and function. Functionality spanned from being ribosome to granule-related, which is sensible as protein analyses are limited to day 6–9 in which neutrophils are no longer dividing and in the final stages of expression would still increase at the final stages of neutrophil maturation ([36, 37]). In comparing the protein expression to transcript expression, we saw that the trends were relatable (Fig 4b).

### Discovery-based data interrogation and network mapping

**Fig 5:**
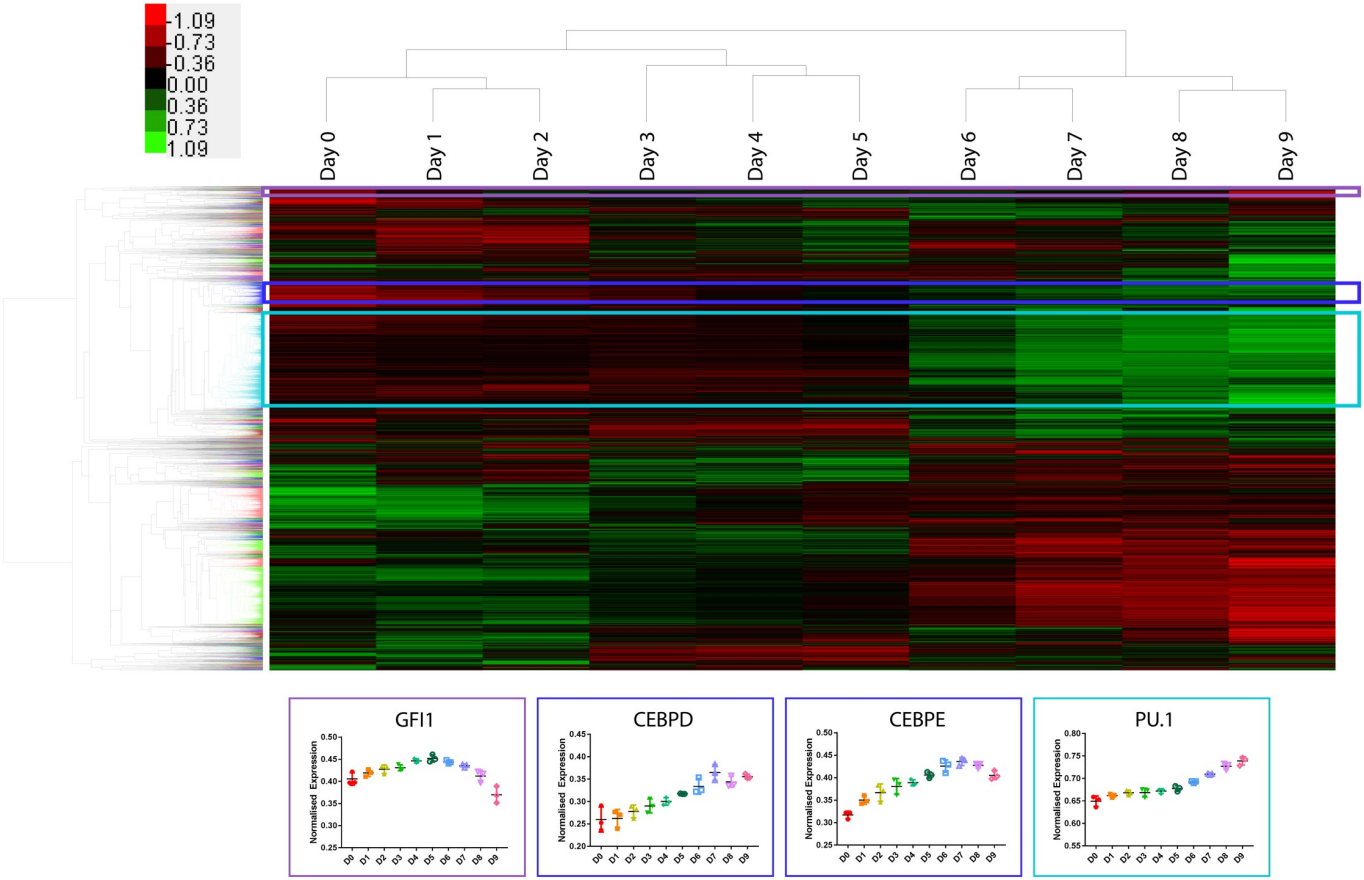
System-wide hierarchical Euclidean distance average linkage clustering shown as a hierarchical clustering of genes. Genes were hierarchically clustered, and the expression profiles (as row-normalized Z scored) are show in a heat map. The associated dendrogram shows sample sub-clustering of days 0–2, days 3–5, and days 6–9. Colouring of the gene dendrogram denotes genes that clustered above 80%. GO-term enrichment analyses were run for genes that clustered with known master regulators of the neutrophil differentiation: *MEIS1* (purple), *CEBPD*,*E* (blue), *PU*.*1* (light blue). Through using literature to manually curate genes within these clusters, several relationships arose that may infer co-regulation. The heatmap is hierarchically clustered based on Euclidean distance and average linkage. Prior to clustering, both genes and arrays were median centered and then normalized such that the sum of the squares of the row and column values were approximately 1.

Ampliseq provided means as an exploratory approach to define regulatory factors governing neutrophil differentiation. As a method to discover transcriptional regulators that may provide insight into the neutrophil differentiation pathway, we used a combinatorial approach of hierarchical clustering, enriched transcription factor binding motifs, and network mapping. An Average-Euclidean distance measure was used for gene and array clustering [18]. The result of the hierarchical Euclidean distance average linkage clustering calculation was displayed in a heatmap as a gene and sample dendrogram with coloring to represent an 80% correlation threshold [19]. Three clusters were identified containing known transcriptional regulators in granulocyte maturation: *GFI1* (82%, purple), *CEBPE*/*CEBPD* (80.8%, dark blue), and *PU*.*1* (80.5%, light blue) (Fig 5). The genes clustered with these known regulators at 80% or higher were analyzed for GO-term enrichment (S2 and S3 Figs).

GO Enrichment analyses were run on clusters containing *PU*.*1*, *CEBPE*, *CEBPD*, and *GFI1* [20]. Revigo was used to visualize the GO enrichment analysis results with the box colour referring to GO category and size referring to enrichment score (S3 and S4 Figs) [21].

Among the 2552 genes that clustered with *PU*.*1* at >80%, we identified 215 significantly enriched (Bonferroni-adjusted P<0.05) GO-terms that grouped into 40 GO-categories (S3 Fig). Highest representation was in the GO categories: leukocyte degranulation and extrinsic apoptotic signaling pathway. Leukocyte degranulation is a key element of neutrophil’s means of host protection as granules are released as a mechanism of host defense [38]. The apoptotic signaling pathway is important in neutrophil development, shown to even be variably expressed in vivo when released from the bone marrow as an inflammatory response [39]. Neutrophil maturation depends on *PU*.*1* for processes such as cell surface protein expression (*CD11b*/*GCSFR*), superoxide production and secondary and tertiary granules development, the latter being key in a degranulation defense response [40, 41]. The cell surface receptors *CD11b* via **β**_2_ integrin interaction as well as *GCSFR* have been linked to apoptotic signaling pathways by relaying signals that delay apoptosis and promote neutrophil survival [42, 43]. This supports the GO-term classification of components that highly clustered with *PU*.*1*.

In the *CEBPE/D* clustering at >80%, 543 components were significantly enriched (Bonferroni-adjusted P<0.05) in 89 GO-Terms spanning 24 GO-Categories (S4 Fig). The largest GO-Category was again leukocyte degranulation, containing 68% of the GO-Terms. CEBPE and CEBPD are key regulators of secondary granule expression [44]. In particular, CEBPD was most effective in regulating *MMP8*—neutrophil collagenase, a secondary granule protein. CEBPE is required for neutrophil maturation past the promyelocyte-myelocyte stage, regulating secondary and tertiary granule formation. In this study, *MMP8* expression is upregulated at day 5 (Fig 3b), suggesting a progression of neutrophil differentiation into the final maturation stages at this transition point.

The components within the *GFI1* cluster were not enriched in GO-terms. However, *GFI1* was highly correlated at 94% with many histones (S5 Fig). GFI1 is known to regulate gene expression through chromatin modulation by co-factor recruitment to histones [45]. Interactions with master transcriptional regulators such as *PU*.*1* and *CEBPE* have been linked to influence hematopoietic differentiation [46].

### Identification of potential gene regulatory networks from Ampliseq data

**Fig 6:**
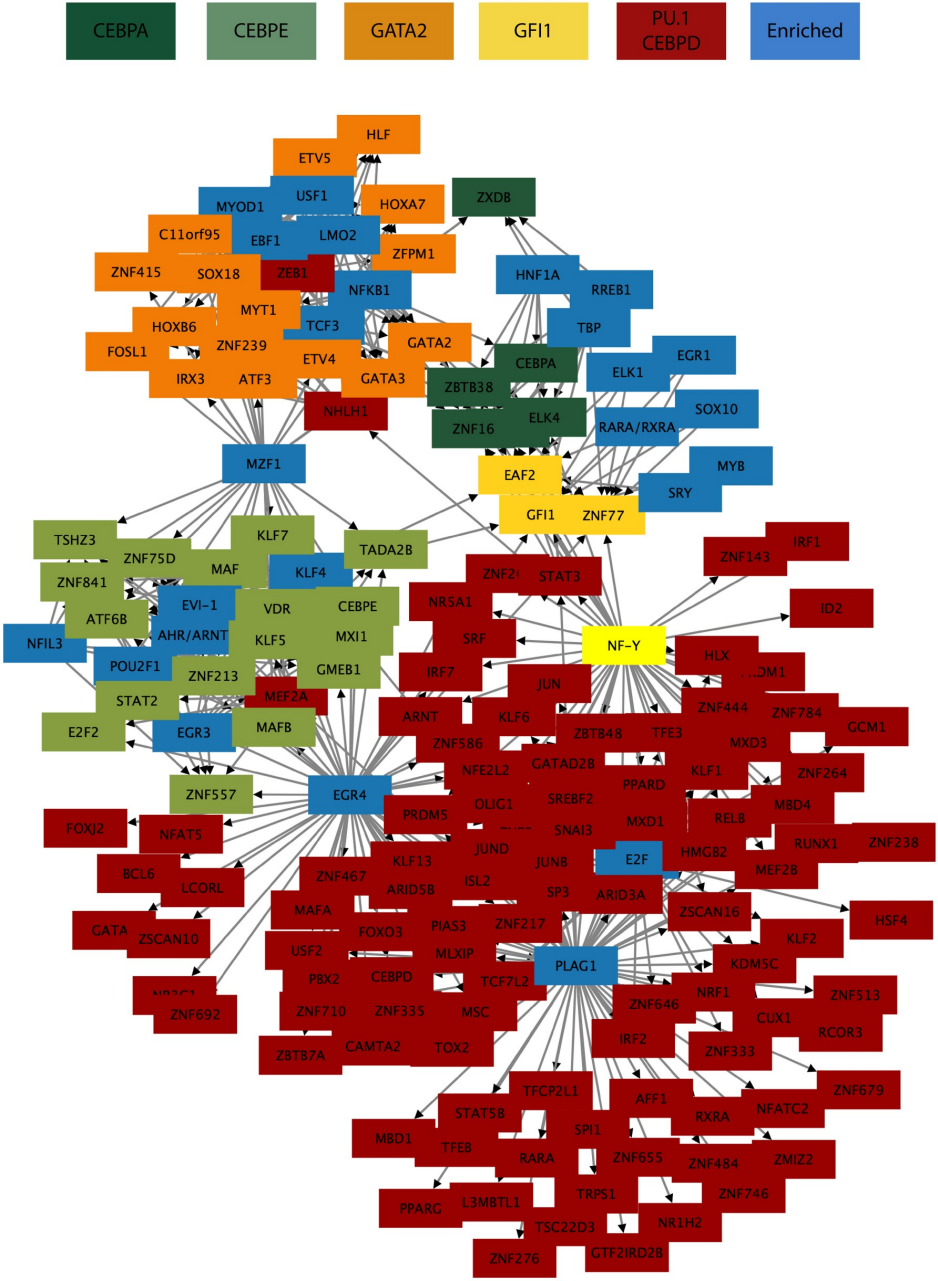
TF binding motif enrichment. Clusters are of TF binding motif enrichment between TFs with expressional correlation clustering at >80% using average-Euclidean distance hierarchical clustering. Universal transcriptional activators such as *SP1* were removed for clarity.

Starting with a list of transcription factors of interest (PU.1, GATA2, GFI1, CEBPA, CEBPD, CEBPE) we investigated the Ampliseq data to infer potential patterns of gene regulation during granulocyte differentiation. The average-Euclidean distance hierarchical clustering was repeated using only the transcription factors identified in the dataset (989 in total). The components that clustered above 80% with our genes of interest listed above were then analyzed using TFM-Explorer [47] for enrichment of transcription factor binding motifs. Proposed interactions between transcription factors identified from the clustering and binding motif analysis were mapped using Cytoscape to generate a targeted potential gene regulatory network involved in granulocyte differentiation (Fig 6). This network contains many known regulators of neutrophil differentiation, including PU.1, CEBPA, CEBPE, CEBPD, and GFI1 confirming the validity of our data and analysis methods.

Among the many well-known transcription factors, we also were able to identify transcription factors involved in the network which are lesser-known or have poorly defined roles in granulopoiesis. These include *ARNT*, present in the *PU*.*1*/*CEBPD* cluster and with a binding motif present in 14 of 21 transcription factors in the *CEBPE* cluster, as well as *NHLH1*, also present in the *PU*.*1*/*CEBPD* cluster and with a binding motif present in 10 of 17 transcription factors in the *GATA2* cluster. Also of interest was the presence of a *PLAG1* binding motif in the regulatory region of 73 of 141 transcription factors in the *PU*.*1*/*CEBPD* cluster.

Aryl hydrocarbon receptor nuclear translocator (ARNT) is a member of the bHLH/PAS nuclear receptor family which dimerises with aryl hydrocarbon receptor (AHR) to form a transcription factor complex [48]. ARNT/AHR signalling has traditionally been identified with xenobiotic response [49], however some studies have identified possible functions for AHR signalling in hematopoietic cell proliferation and differentiation, including stem cell maintenance [50], erythroid/megakaryocyte differentiation [51], and acute inflammatory response [52]. Whether or not these roles are ARNT-dependent remains to be seen as AHR has been shown to dimerise with other non-canonical binding partners [49]. However, ARNT fusion products have been described in leukemias, including AML [53]. In this case, it was hypothesised that expression of the described TEL-ARNT fusion product may have interfered with normal ARNT function, contributing to the development of AML. Our data shows a slight upward trend in expression of *ARNT* over the differentiation time course, fitting with the increasing expression of *CEBPE* cluster genes, indicating this factor is an attractive candidate for further evaluation (S6 Fig).

NHLH1 (NSCL1, HEN1) is a member of the basic helix-loop-helix (bHLH) family of transcription factors expressed in the developing nervous system and involved in neuronal differentiation [54, 55]. Despite being highly homologous to erythroid differentiation factor SCL [56], NHLH1 was shown not to interact with typical SCL binding partners and does not appear to be involved in erythroid differentiation [57]. Rather, our expression data shows an increase in expression in the latter stages of neutrophil differentiation (S6 Fig). When comparing this expression pattern to the GATA2-cluster genes containing a NHLH1 binding motif, however, it is difficult to see how these factors might relate to each other. Although both *GATA2* and *GATA3* showed increased expression at the final time points, for most of the genes in the cluster this was not the case. Due to the similarity between NHLH1 and SCL, it is possible that these genes in the GATA2 cluster instead contain non-canonical binding motifs for SCL which are being erroneously attributed to NHLH1. In fact, SCL is known to bind to the promoter regions of multiple genes in this cluster [12, 58, 59]. Regardless of our confidence in the motif analysis, the expression pattern of *NHLH1* which resulted in clustering with *PU*.*1* and *CEBPD*, raises the possibility of this factor being involved in the later stages of granulocyte differentiation.

PLAG1 is a known oncogene first identified in pleomorphic adenomas of the salivary gland, and later found to be upregulated in CLL due to deregulation of repressive miRNAs [60, 61]. A study showing cooperativity with a translocation product in AML demonstrated that it exerts a strong proliferative effect by inducing the G1-S transition causing enhanced expansion and cell renewal [62]. Recently it has been suggested that *PLAG1* expression in the hematopoietic system is restricted to the most primitive stem cells, with the authors proposing that it might be a novel regulator of primitive stem cell self-renewal and proliferation [63]. Although the gene itself was not expressed in this dataset obtained from cells further down the differentiation hierarchy, the presence of this motif across so many granulocyte-linked genes hints that part of the effect of *PLAG1* on stem cell expansion may be modulated through repression of transcription of lineage-specific genes.

These examples of unfamiliar factors present in our gene regulatory network indicate areas where further analysis could potentially result in enhanced understanding of the differentiation process and shows the utility of the Ampliseq platform for investigating such questions.

### Examination of non-coding RNA expression in neutrophil differentiation

In line with our assertion that the transcriptional networks comprising the middle stages of neutrophil differentiation are not well defined, there is currently little known about the involvement of non-coding RNA in this process. We hypothesised that we could use our data to uncover previously unannotated roles for non-coding RNA at different stages of neutrophil development. Finding other RNAs with similar expression profiles to myeloid differentiation factors, for instance, may provide a list of possible targets to explore further.

Use of clustering as described above allowed us to identify non-coding RNAs that displayed expression trends similar to factors of interest in our differentiation process. Non-coding RNAs clustering with *PU*.*1* and showing similar expression profiles included *ITGB2-AS1*, *FLJ39051*, *LOC100289473*, *LOC100505622*, *TREML3P*, *LEMD-AS1* (S7 Fig). Further investigation of this list found two of these transcripts have previously been implicated in myeloid differentiation.

Non-coding RNA *FLJ39051*, also known as *GSEC* (G-quadruplex-forming sequence containing lncRNA [64]), clustered closely with *PU*.*1* and shared similar expression profiles across the experimental time series. GSEC has recently been shown to directly interact with and inhibit the RNA helicase DHX36 (DEAH box polypeptide 36) in a colon cancer cell line [64]. DHX36 has been implicated in leukemia through interaction with apoptosis and caspase activation inhibitor, AVEN, to increase translation of leukemogenic transcriptional regulators MLL1 and MLL4, and subsequent proliferation of leukemic cells [65]. Given this, it is reasonable to speculate that GSEC may play a role in down-regulation of proliferation genes through inhibition of *DHX36* as cells progress further along the differentiation pathway.

Upon close inspection, *LOC100289473* appears to be transcribed from the sequence identified as *SIRPβ3p* as described in a study using gene prediction based on sequence similarity and analysis of genomic regions containing known *SIRP* genes in various species [66]. The SIRP (signal-regulatory protein) family members are transmembrane glycoproteins with diverse function in immune cell regulation expressed mainly by myeloid cells [67]. The SIRPβ3p gene in humans was designated a pseudogene, despite computational predictions of the existence of an open reading frame and suggestion of functional expression from rodent orthologs, due to a lack of evidence for expression in humans [67]. Our data may provide some evidence that this gene is indeed transcribed in human and potentially has a role in myeloid function.

The appearance of these two ncRNA transcripts on our list of interest suggests that Ampliseq could allow identification of uncharacterised non-coding RNAs which may potentially be biologically relevant in this system and warrant further investigation.

In the list of non-coding RNAs clustering with *PU*.*1*, we also identified a number of other transcripts displaying a very particular expression profile characterised by initial steady, low expression followed by a strong increase in expression from day 6 onwards. This time frame aligns with the transition from the myelocyte to the nondividing, metamyelocyte phenotype and therefore may be the point in differentiation in which cells stop dividing and begin the completion of the differentiation process through final morphological development. These transcripts included *LOC606724*, *NEAT1*, *LOC729737*, *FLJ45340*, *LOC100133161*, and *MIAT*, and their profiles are shown in S8 Fig.

One of these transcripts, the long non-coding RNA *NEAT1*, has been reported in the literature as overexpressed in several types of solid tumour [68]. In contrast, inhibition of *NEAT1* has been shown to impair myeloid differentiation in promyelocytic leukemia cells [69]. This fits with our data showing an increase of expression of this transcript as cells are entering the terminal stages of differentiation. Again, this list may provide candidates for further validation of participation in terminal neutrophil differentiation.

## Discussion

In an era where gene editing is a reality, the question of “what to edit” remains unanswered. This is mainly because cellular functions arise from interacting networks of genes and their regulators rather than simple single causes. Therefore, a deeper understanding of gene networks and their evolving landscape during differentiation is required. This is not trivial given the vast network variability caused by slight changes in the expression of master gene regulators during differentiation. Moreover, cell population heterogeneity in lineage potential, priming, and development as well as the wide spread of cells’ quiescence state enhances even further the variability of gene expression within the networks. As a result, gene expression data are inherently variable making it difficult to draw simple inferences that link gene regulatory mechanisms to differentiation programs in myeloid transient populations.

In response, we have applied a novel approach to surmount these challenges in a study of the neutrophil differentiation pathway to identify differentiation regulators using a transcriptome resequencing assay (Ampliseq RNA) supported by ddPCR and discovery mass spectrometry proteomics. Within this framework, Ampliseq allowed for a broad transcriptomic discovery-based approach and was advantageous in accommodating minimal input material, which represents a major hurdle when working with low numbers of primary cells derived from umbilical cord blood. This allowed for regular time series sampling from a neutrophil differentiation with a limited initiating cell population and provided the opportunity to assess the capacity of Ampliseq to identify novel regulatory transcription factors in a biologically relevant system.

Transcriptional regulators driving neutrophil development are believed to serially up-regulate one another, resulting in a deterministic process [12]. In an *ex vivo* culturing system, we were able to increase homogeneity by using actively proliferating (pre-cultured) granulocyte-primed progenitors that were sourced from the same umbilical cord sample. The 24-hour sampling intervals across 9 days of the differentiation, resulted in a high resolution, time-series, Ampliseq dataset which allowed to discern patterns in the gene expression networks. From these patterns, inferences could then be drawn as to regulatory relationships between transcription factors, and also identified ncRNAs as being involved in this process. Such proposed relationships provide a step towards gaining a more comprehensive regulatory map to understand the neutrophil differentiation process.

Capturing and replicating expression patterns of known, highly expressed components in the differentiation pathway were used as a baseline check for Ampliseq. A combination of known cell surface receptors, granules, and master regulator transcription factors were used for this first assessment [70]. A clear pattern of granule development showed the expected expression patterns of primary, secondary and tertiary granules (Fig 3b). Additionally, the usual pattern of serially upregulated cell surface proteins were captured by the Ampliseq system (Fig 3a). *GCSFR* was consistently expressed and *CD11b* started to increase in expression around day 4, perhaps marking the promyelocyte/myelocyte stages [70].

While transcriptional regulators are comparatively lowly expressed to these receptor and granule proteins, we selected two known transcription factors with opposite expression patterns in the granulocyte differentiation pathway, *PU*.*1* and *MEIS1* for further validation testing using digital PCR. The expression patterns between digital PCR and Ampliseq matched, proving that Ampliseq is a capable of elucidating known patterns as a discovery-based approach.

Proteomics data supported transcript expression trajectories; however, approximately 1 x 10^6^ cells were required for reliable results and therefore, expression trends from only the last 4 days were used for analyses. The top variable proteins (coefficient variation >50%) from SWATH analysis had distinctive increasing or decreasing trends. These protein expression trends were matched by the transcriptome data. Three of the proteins that were increasing in expression trajectory with time, SA1008/9 and 12, are important regulators of the inflammatory response, and thus expected to increase with neutrophil maturation.

Ampliseq data was clustered using a Euclidean distance average linkage metric and clusters formed at >80% correlation and containing known granulocyte transcriptional regulators (*PU*.*1*, *CEBPE*, *CEBPD*, and *GFI1*) were analyzed for GO-term enrichment. *GFI1*, known in chromatin remodelling, highly clustered with histones. *PU*.*1* and *CEBPE/D* were both most enriched in the leukocyte degranulation GO-category, a primary component of neutrophil’s functionality in host protection.

We were interested in finding patterns of regulators that may up-regulate or down-regulate transcription factors during the neutrophil differentiation trajectory. As causality cannot be inferred from transcriptome analyses, we explored correlative relationships. Gene and array clustering were explored for both the complete dataset and for a subgroup containing only transcription factors. By applying a hierarchical Euclidean distance average linkage clustering method on the complete dataset, we were able to isolate genes that clustered above 80% with known granulocyte transcription factors and further analyze these subgroupings for GO-term enrichment. The significantly enriched factors within the GO-term enrichment (Bonferroni-adjusted P<0.05) were confirmed to be relevant to the subcategorizing transcription factors. The same Euclidean distance average linkage clustering method analysis was run for transcription factors to identify potential gene regulatory networks. Regulators that clustered above 80% with known granulocyte TFs (*PU*.*1*, *CEBPA*, *CEBPE*, *CEBPD*, and *GFI1*) were then analyzed using TFM-Explorer [47] for enrichment of transcription factor binding motifs. Mapping these factors in Cytoscape identified *ARNT* and *NHLH1* as having binding motifs present in *PU*.*1*/*CEBPD* and *GATA2* clusters, respectively. The expression trends of these regulators represented that of the transcriptional regulators that they were linked to through binding motifs, suggesting regulatory relationships.

*PLAG1*, an early expressed factor in the dataset, was another highlighted factor that was gained from the exploratory analyses. The *PLAG1* motif was present across many granulocyte-linked genes and suggests an effect on stem cell expansion, which may be modulated through repression of transcription of lineage-specific genes. As a known oncogene, finding such correlations may provide important information in guiding downstream experimental efforts.

The regulatory role of non-coding RNAs are gaining deserved attention and we focused some of our discovery-based analyses on uncovering RNAs with similar expression profiles to myeloid differentiation factors. We used the same clustering method as described above to identify non-coding RNAs that clustered with known granulocyte transcriptional regulators. Several non-coding RNAs clustered in sharing an expression profile of being stably lowly expressed prior to a clear increase after day 6. Included in this list, a previously reported RNA, *NEAT1*, were identified and had an expression profile that matched literature suggested functions. Several lesser-known factors were extracted as well, e.g. *GSEC* closely clustered with *PU*.*1*, and through literature review is suggested to have an interactive role in the regulation of proliferation-based genes. These factors may provide candidates for further validation of participation in terminal neutrophil differentiation.

This study has demonstrated that Ampliseq provides a new competitive discovery-based technique for whole transcriptome sequencing. Mass spectrometry based proteomics and ddPCR assays were also employed to validate numerous Ampliseq derived expression profiles. Through correlating expression profiles to known transcription factors and then building maps of the suggested correlative relationships, we developed a list of possible targets for further exploration. Although this study was limited by the use of a single biological sample (i.e., only cells derived from a single cord blood donor were evaluated), we can confidently conclude that the technical reproducibility of the methods examined were sufficiently robust. In the age of genome editing, the downstream differentiation map is a valuable comparison standard as we aim to derive factors for directing phenotype and expansion enhancement.

## Supporting information

S1 Fig HK transcriptomic expression and proteomic expression. Protein HK expression was extremely low on days 1–5 due to low cell number/sample. Therefore, only proteomic samples 6–9 were considered for further data set analyses. The heatmap values are plotted post-DESeq2 normalization and as the rlog transform to a scale between 0 and 1, retaining rank order and the relative size of separation between values.
(TIF)

S2 Fig Correlation of ddPCR and Ampliseq RNA data. (A) digital PCR was used as a validation approach for 2 reference genes (ACTN4 and POL2). The expression profiles for each target overlap for each method: Ampliseq and ddPCR, supporting Ampliseq as a sound method for transcriptome analyses. ACTN4 showed intermediate and highly stable expression values. POL2 was lowly expressed, resulting in higher variability amongst triplicates, but a maintained mean expression level across D0 to D9. (B) Scatterplot comparison of ddPCR and Ampliseq RNA expression values plotted against each other. The trendline R^2 value is also included in the graph, indicating a strong linear correlation between the two methods for the selected genes. (C) Ampliseq sequencing read counts per sample.
(TIF)

S3 Fig PU.1 cluster GO enrichment analyses. The 2552 genes clustered with PU.1 at a correlation of 80.4% were enriched in 40 GO-categories across 215 GO-terms.
(TIF)

S4 Fig CEBPE/D cluster GO enrichment analyses. The 543 genes clustered with CEBPE/D at a correlation of 80.9% were enriched in 14 GO-categories across 51 GO-terms.
(TIF)

S5 Fig GFI GO-term enrichment clusters closely with histones. GFI1 correlates with 75 genes at 82.2%. While no GO-terms were enriched for this broader cluster, GFI1 was nested within 14 histones, at a high correlation of 94%. GFI1 is known for down-regulation of gene expression through co-factor recruitment [33]. The heatmap values are plotted post-DESeq2 normalization and as the rlog transform to a scale between 0 and 1, retaining rank order and the relative size of separation between values.
(TIF)

S6 Fig Correlated transcription factor expression profiles. ARNT and NHLH1, both present in the PU.1-CEBPD cluster, had enriched binding motifs in TFs clustering with CEBPE and GATA2 at >80%. ARNT had a binding motif present in 14 of 21 factors in the CEBPE cluster and NHLH1 had a binding motif present in 10 of 17 factors in the GATA2 cluster.
(TIF)

S7 Fig Identified ncRNAs that clustered >80% with PU.1. A selection of ncRNAs that clustered above 80% with PU.1 showed a similar upward expression trajectory: ITGB2-AS1, FLJ39051, LOC100289473, LOC100133331, LOC100505622, LOC100499194, TREML3P, LEMD-AS1. As not much information is known on ncRNAs during the neutrophil maturation process, these targets may serve as initial targets for further investigation.
(TIF)

S8 Fig Identified ncRNAs that clustered >80% with PU.1 and had increased expression profiles towards the end of the differentiation process. A selection of ncRNAs that clustered above 80% with PU.1 showed an unusual expression trajectory with a relatively flat expression profile for the first five days, followed by a jump of increased expression: LOC606724, NEAT1, LOC729737, FLJ45340, LOC100133161, and MIAT. As not much information is known on ncRNAs during the neutrophil maturation process, these targets may serve as initial targets for further investigation.
(TIF)

S1 File (CSV)

S2 File (XLS)

S3 File (XLS)

S1 References (DOCX)
