# Supplementary figures and images for: Network mapping of primary CD34+ cells by Ampliseq based whole transcriptome targeted resequencing identifies unexplored differentiation regulatory relationships
Source: PLoS One. 2021 Feb 5;16(2):e0246107. doi: 10.1371/journal.pone.0246107 (PMC7864404; doi:10.1371/journal.pone.0246107)

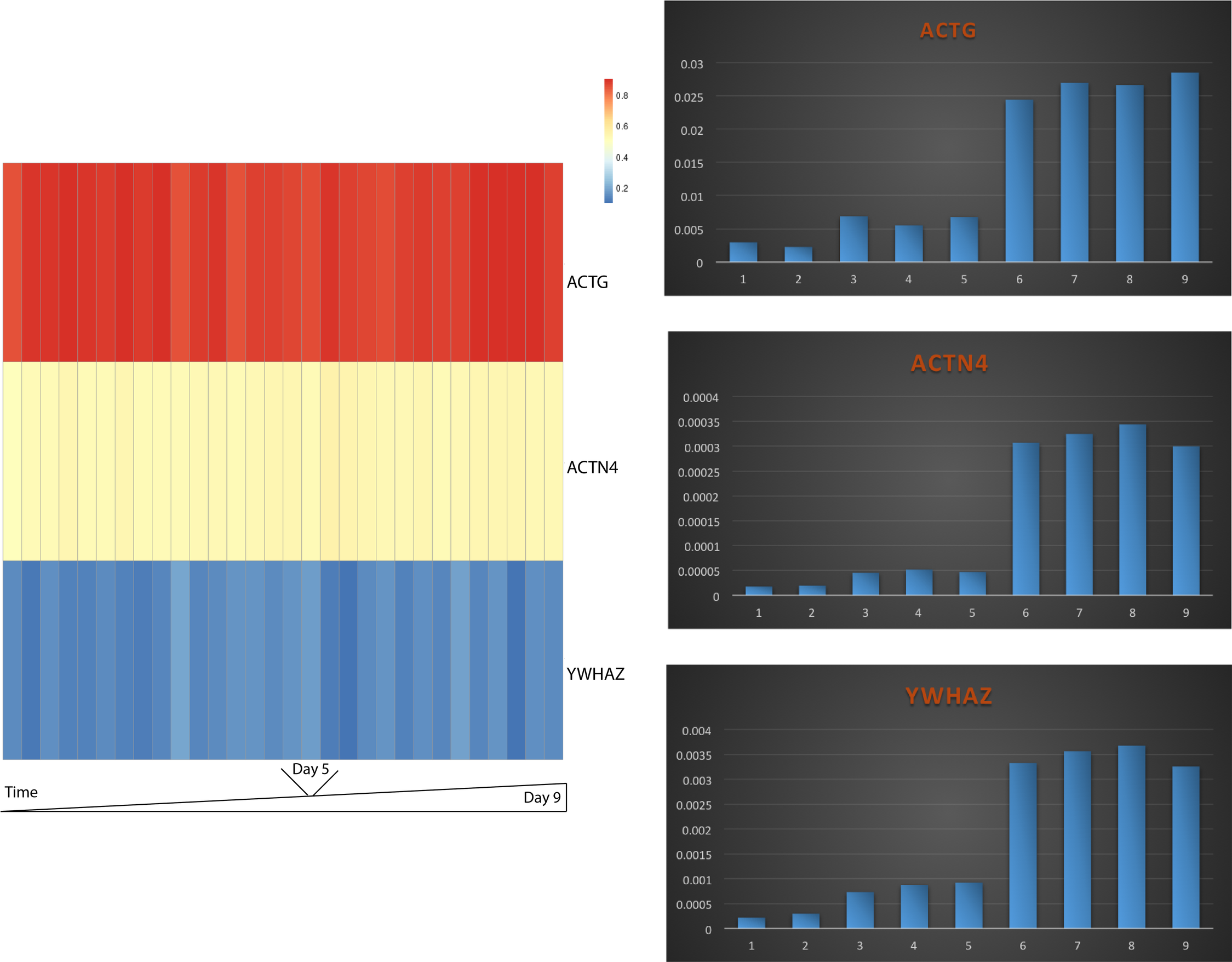

Supplement: S1 Fig — Protein HK expression was extremely low on days 1–5 due to low cell number/sample. Therefore, only proteomic samples 6–9 were considered for further data set analyses. The heatmap values are plotted post-DESeq2 normalization and as the rlog transform to a scale between 0 and 1, retaining rank order and the relative size of separation between values. (TIF) [file pone.0246107.s001.tif]

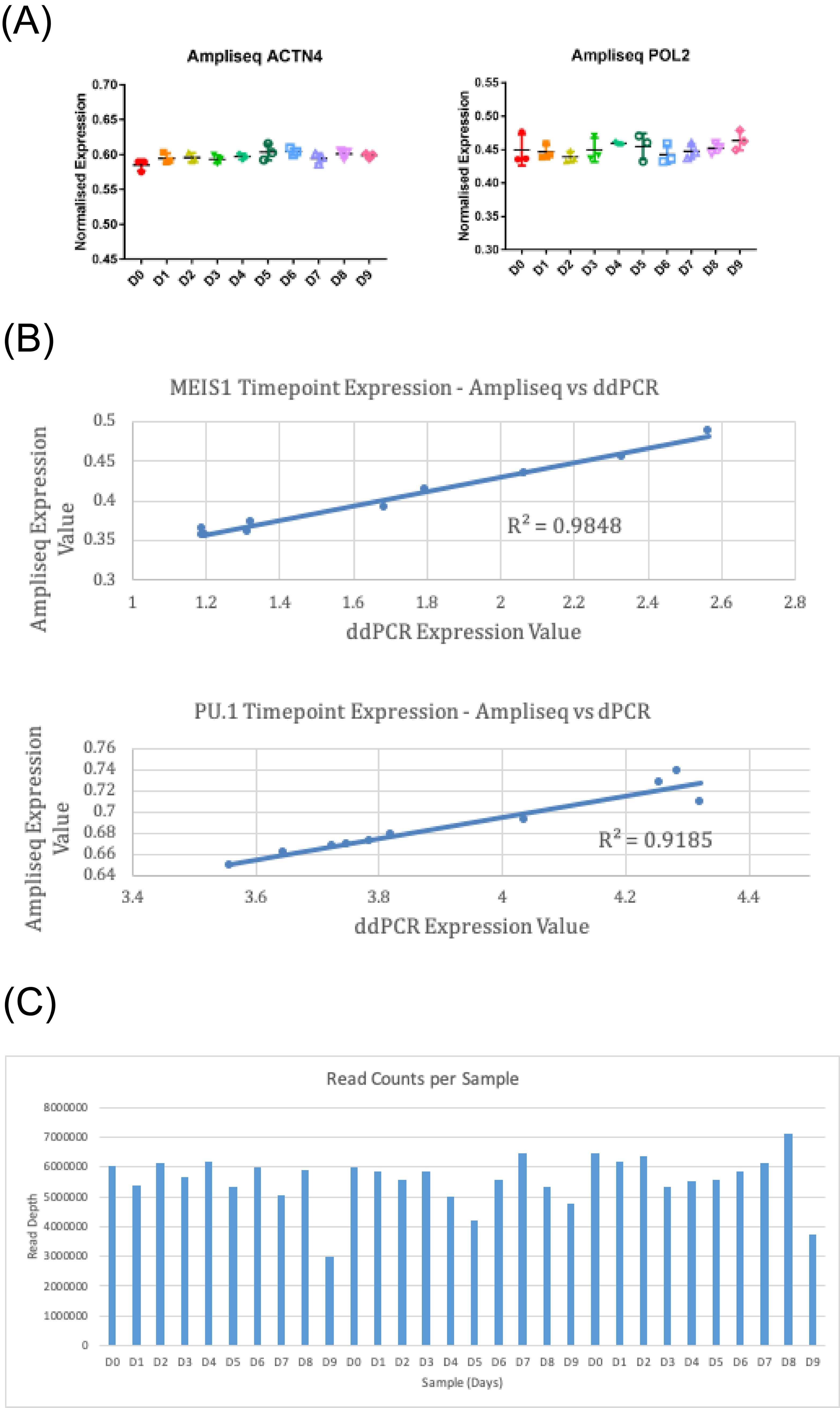

Supplement: S2 Fig — (A) digital PCR was used as a validation approach for 2 reference genes (ACTN4 and POL2). The expression profiles for each target overlap for each method: Ampliseq and ddPCR, supporting Ampliseq as a sound method for transcriptome analyses. ACTN4 showed intermediate and highly stable expression values. POL2 was lowly expressed, resulting in higher variability amongst triplicates, but a maintained mean expression level across D0 to D9. (B) Scatterplot comparison of ddPCR and Ampliseq RNA expression values plotted against each other. The trendline R^2 value is also included in the graph, indicating a strong linear correlation between the two methods for the selected genes. (C) Ampliseq sequencing read counts per sample. (TIF) [file pone.0246107.s002.tif]

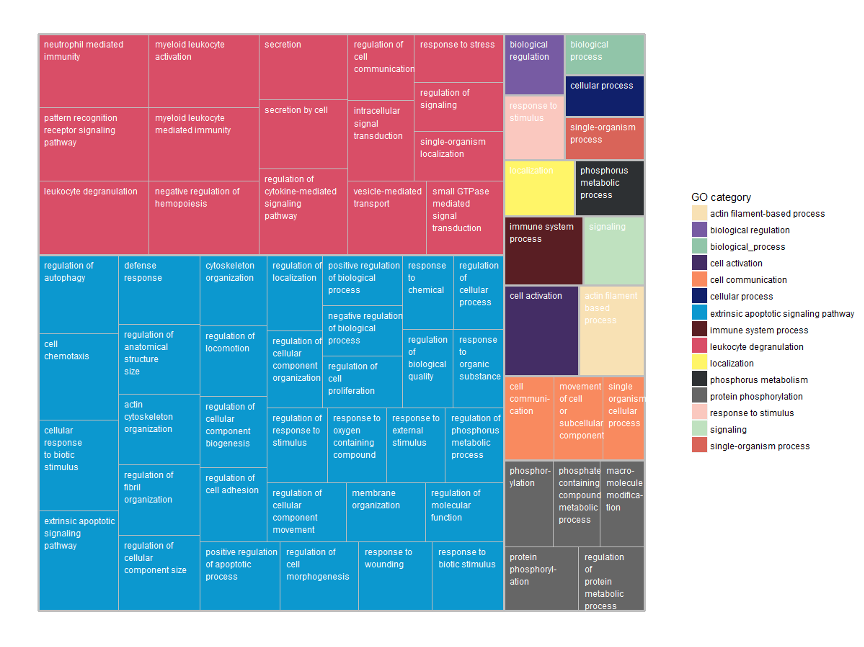

Supplement: S3 Fig — The 2552 genes clustered with PU.1 at a correlation of 80.4% were enriched in 40 GO-categories across 215 GO-terms. (TIF) [file pone.0246107.s003.tif]

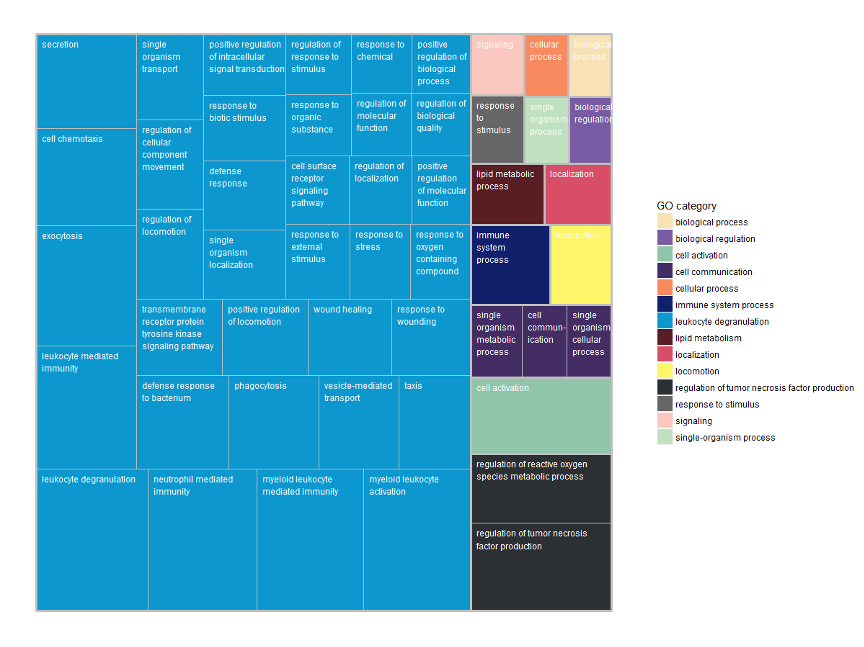

Supplement: S4 Fig — The 543 genes clustered with CEBPE/D at a correlation of 80.9% were enriched in 14 GO-categories across 51 GO-terms. (TIF) [file pone.0246107.s004.tif]

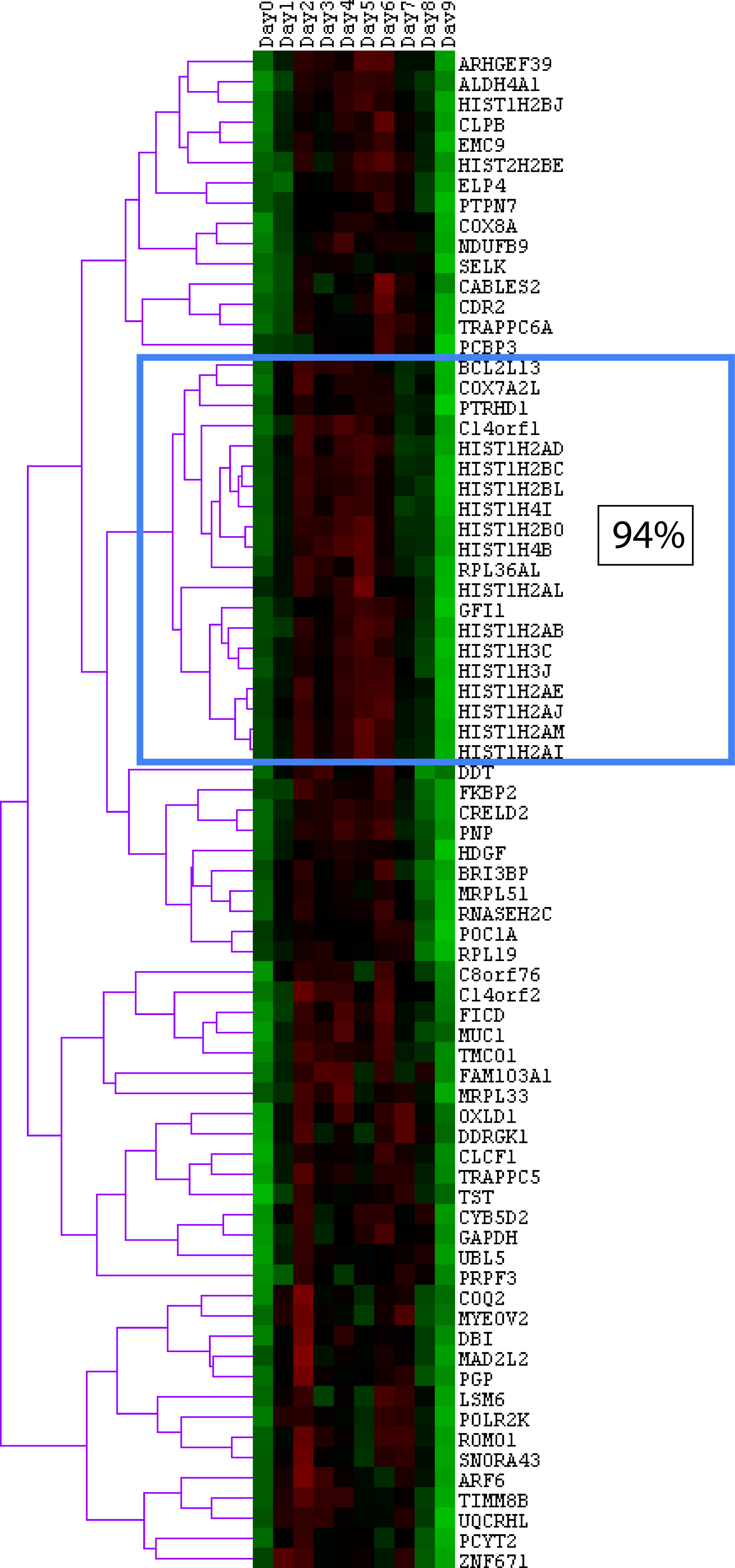

Supplement: S5 Fig — GFI1 correlates with 75 genes at 82.2%. While no GO-terms were enriched for this broader cluster, GFI1 was nested within 14 histones, at a high correlation of 94%. GFI1 is known for down-regulation of gene expression through co-factor recruitment [33]. The heatmap values are plotted post-DESeq2 normalization and as the rlog transform to a scale between 0 and 1, retaining rank order and the relative size of separation between values. (TIF) [file pone.0246107.s005.tif]

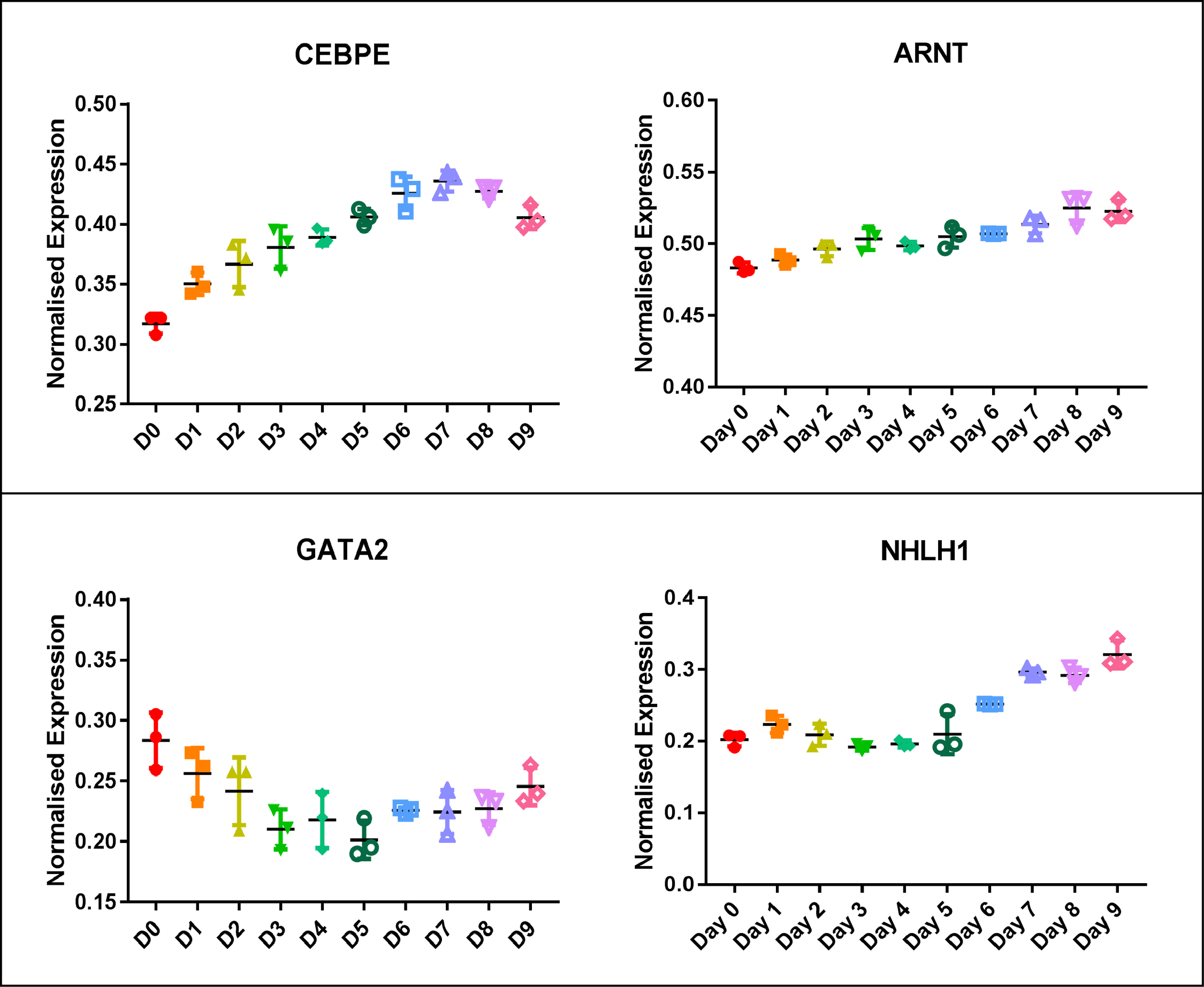

Supplement: S6 Fig — ARNT and NHLH1, both present in the PU.1-CEBPD cluster, had enriched binding motifs in TFs clustering with CEBPE and GATA2 at >80%. ARNT had a binding motif present in 14 of 21 factors in the CEBPE cluster and NHLH1 had a binding motif present in 10 of 17 factors in the GATA2 cluster. (TIF) [file pone.0246107.s006.tif]

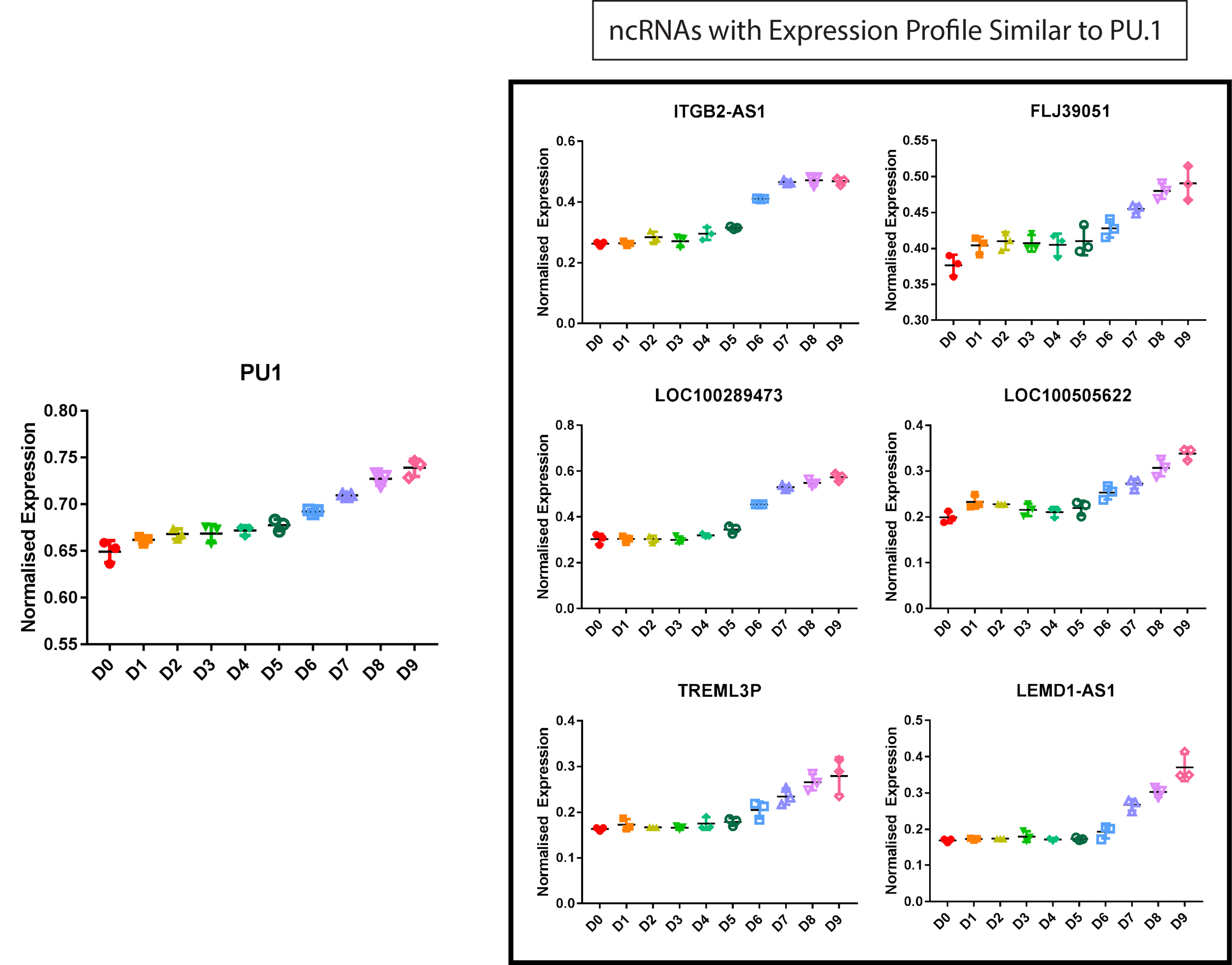

Supplement: S7 Fig — A selection of ncRNAs that clustered above 80% with PU.1 showed a similar upward expression trajectory: ITGB2-AS1, FLJ39051, LOC100289473, LOC100133331, LOC100505622, LOC100499194, TREML3P, LEMD-AS1. As not much information is known on ncRNAs during the neutrophil maturation process, these targets may serve as initial targets for further investigation. (TIF) [file pone.0246107.s007.tif]

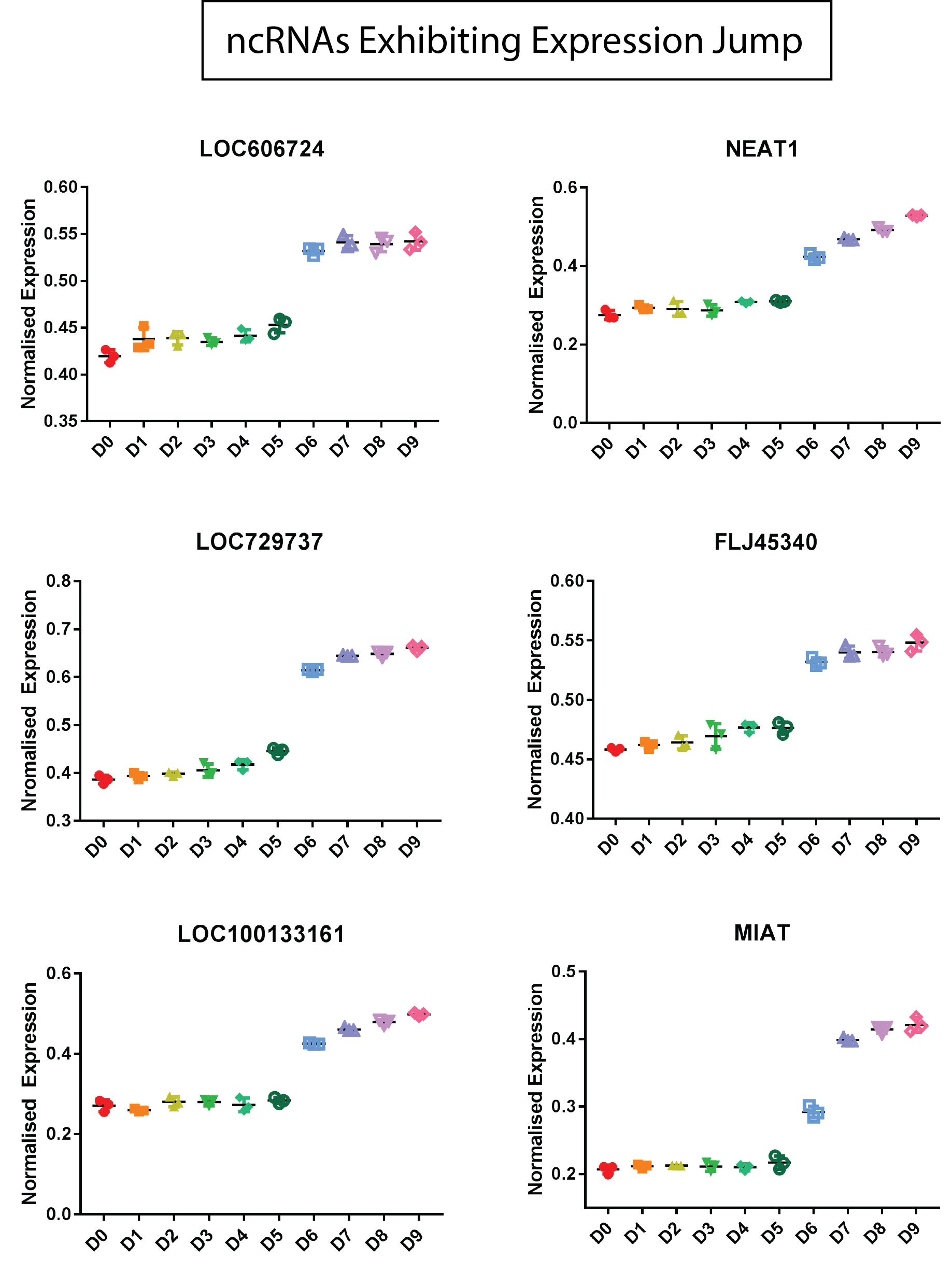

Supplement: S8 Fig — A selection of ncRNAs that clustered above 80% with PU.1 showed an unusual expression trajectory with a relatively flat expression profile for the first five days, followed by a jump of increased expression: LOC606724, NEAT1, LOC729737, FLJ45340, LOC100133161, and MIAT. As not much information is known on ncRNAs during the neutrophil maturation process, these targets may serve as initial targets for further investigation. (TIF) [file pone.0246107.s008.tif]
