# Supplementary material for: Network mapping of primary CD34+ cells by Ampliseq based whole transcriptome targeted resequencing identifies unexplored differentiation regulatory relationships
Source: PLoS One. 2021 Feb 5;16(2):e0246107. doi: 10.1371/journal.pone.0246107 (PMC7864404; doi:10.1371/journal.pone.0246107)
Supplement: S1 References — (DOCX) [file pone.0246107.s012.docx]

S1 References

1. Gene Ontology C. Gene Ontology Consortium: going forward. Nucleic acids research. 2015 Jan;43(Database issue):D1049-56. PubMed PMID: 25428369.
2. Supek F, Bosnjak M, Skunca N, Smuc T. REVIGO summarizes and visualizes long lists of gene ontology terms. PloS one. 2011;6(7):e21800.
